# Supplementary material for: Agreement between commercially available ELISA and in-house Luminex SARS-CoV-2 antibody immunoassays
Source: Sci Rep. 2021 Sep 23;11:18984. doi: 10.1038/s41598-021-98296-y (PMC8460676; doi:10.1038/s41598-021-98296-y)
Supplement: Supplementary file 1 — Supplementary Table S1. [file 41598_2021_98296_MOESM1_ESM.pdf]

TITLE

Agreement between commercially available ELISA and in-house Luminex SARS-CoV-2 antibody immunoassays

AUTHORS

Rebeca Santano[1]*, Diana Barrios[1], Fàtima Crispi[2], Francesca Crovetto[2], Marta Vidal[1], Jordi Chi[1], Luis Izquierdo[1], Eduard Gratacós[2], Gemma Moncunill[1], Carlota Dobaño[1]*

INSTITUTIONS

[1]ISGlobal, Hospital Clínic - Universitat de Barcelona, Barcelona, Catalonia, Spain

[2]BCNatal, Barcelona Center for Maternal-Fetal and Neonatal Medicine, Hospital Sant Joan de Déu and Hospital Clínic, IDIBAPS, Universitat de Barcelona, CIBER-ER, Barcelona, Spain

*CORRESPONDING AUTHORS

rebeca.santano@isglobal.org

carlota.dobano@isglobal.org

Supplementary Table S1. Performance of ELISA based on Luminex diagnostic as gold standard in samples with more than 15 days since onset of symptoms. The analysis included samples from mothers (N=21). The section of “All isotypes” refers to the performance based on seropositivity by any of the three isotypes. FN: False negative; FP: False positive, TN: True negative; TP: True positive; SE: Sensitivity; SP: Specificity, PPV: Positive predictive value; NPV: Negative predictive value.

Isotype	FN	FP	TN	TP	Total	SE (%)	SP (%)	PPV(%)	NPV(%)
**IgG**	1	0	11	9	21	90	100	100	92
**IgM**	0	9	4	8	21	100	31	47	100
**IgA**	1	8	3	9	21	90	27	53	75
**All isotypes**	0	7	3	11	21	100	30	61	100
